# Methodological survey of designed uneven randomization trials (DU-RANDOM): a protocol

**DOI:** 10.1186/1745-6215-15-33

**Published:** 2014-01-23

**Authors:** Darong Wu, Elie A Akl, Gordon H Guyatt, Philip J Devereaux, Romina Brignardello-Petersen, Barbara Prediger, Krupesh Patel, Namrata Patel, Taoying Lu, Yuan Zhang, Maicon Falavigna, Nancy Santesso, Reem A Mustafa, Qi Zhou, Matthias Briel, Holger J Schünemann

**Affiliations:** 1 Department of Clinical Epidemiology, 2nd Affiliated Hospital of Guangzhou University of Chinese Medicine, Guangzhou, China; 2 Departments of Internal Medicine, American University of Beirut, Beirut, Lebanon; 3 Department of Clinical Epidemiology & Biostatistics, McMaster University, Hamilton, ON, Canada; 4 Departments of Medicine, State University of New York at Buffalo, Buffalo, NY, USA; 5 Institute of Health Policy, Management and Evaluation, University of Toronto, Toronto, ON, Canada; 6 Faculty of Dentistry, University of Chile, Santiago, Chile; 7 Department of Medical Biometry and Statistics, Albert-Ludwigs-University, Freiburg, Germany; 8 Department of Biology (Physiology Specialization), McMaster University, Hamilton, ON, Canada; 9 Epidemiology and Health Technology Assessment Institute, Universidade Federal do Rio Grande do Sul, Porto Alegre, Brazil; 10 Department of Internal Medicine, Division of Nephrology, University of Missouri Kansas City, Kansas City, MO, USA; 11 Basel Institute for Clinical Epidemiology & Biostatistics, University Hospital Basel, Basel, CH, Switzerland

**Keywords:** Participation rate, Designed uneven randomization trials, Trial participation

## Abstract

**Background:**

Although even randomization (that is, approximately 1:1 randomization ratio in study arms) provides the greatest statistical power, designed uneven randomization (DUR), (for example, 1:2 or 1:3) is used to increase participation rates. Until now, no convincing data exists addressing the impact of DUR on participation rates in trials. The objective of this study is to evaluate the epidemiology and to explore factors associated with DUR.

**Methods:**

We will search for reports of RCTs published within two years in 25 general medical journals with the highest impact factor according to the Journal Citation Report (JCR)-2010. Teams of two reviewers will determine eligibility and extract relevant information from eligible RCTs in duplicate and using standardized forms. We will report the prevalence of DUR trials, the reported reasons for using DUR, and perform a linear regression analysis to estimate the association between the randomization ratio and the associated factors, including participation rate, type of informed consent, clinical area, and so on.

**Discussion:**

A clearer understanding of RCTs with DUR and its association with factors in trials, for example, participation rate, can optimize trial design and may have important implications for both researchers and users of the medical literature.

## Background

Well-designed and implemented randomized controlled trials (RCTs) are considered the ‘gold-standard’ for evaluating the effects of health care interventions. However, the recruitment of participants to RCTs can be extremely difficult [1,2]. For example, in a review of 41 RCTs [3], a large proportion (66%) failed to achieve their planned sample size [4-6]. In the same review [3], those who refused to participate accounted for 27% of those eligible, thus limiting the generalizability of the findings.

Patient preferences for a particular treatment option are reported as the top barrier to participation in RCTs [7]; the key reasons for the refusal to participate are usually the rejection of receiving placebo or other inactive therapy [8,9]. Several investigators report [10] that patients who thought that the experimental treatment was better than standard treatment were more likely to consent to participation in RCTs [11,12]. Thus, a greater chance of receiving active treatment rather than placebo or control therapy may lead to greater participation in RCTs.

Encouraging patient enrollment is one of the main reasons for designed uneven randomization (DUR), such as 1:2 or 1:3 or greater [2,13,14]. Other reasons include reducing costs, avoiding presumed risks (for example, toxicity), or increasing the amount of information on safety/adverse events of an intervention [15-17]. A DUR trial can be defined as a trial that is pre-planned to include a different numbers of participants in the experimental and control groups. However, so far, investigators have not comprehensively examined the reasons for conducting DUR trials and the impact of DUR on participation rate.

Treweek *et al*. [18] systematically reviewed randomized and quasi-randomized controlled trials evaluating the effect of strategies to improve recruitment of participants to RCTs. Their results showed that participation rates might be influenced by a number of factors [18] such as study design, consent form, approach made to potential participants or financial incentives for participants, but there was not enough data to rule out or confirm a significant effect of DUR. Two studies [10,19] included in the systematic review evaluated various combinations of pre-randomization and consent. One study recruited 3,217 healthy participants who were predominantly young males with a high level of education. Another was a mock anesthesia trial, in which participants were asked to choose between different anesthetic drugs. The applicability of these results remains uncertain given the hypothetical nature of the exercise [10].

Thus, until now, no convincing data that addresses the impact of DUR on factors, such as participation rates, in trials exists despite the more than anecdotal use and publications of DUR trials in high impact journals [20-25]. The primary objective of this study is to explore factors associated with DUR. The secondary objectives are to describe (1) the epidemiology, (2) the justification for using DUR, and (3) the methodological quality of DUR trials.

## Methods

### Overall study design

**Figure 1:**
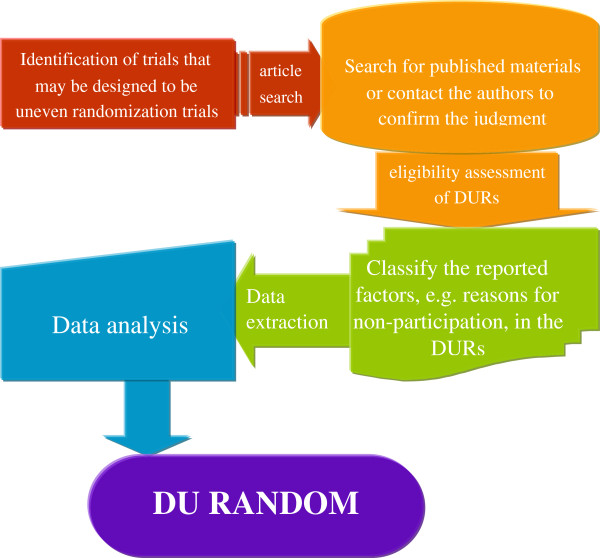
Flow chart of the methodological survey of designed uneven randomization trials (DU-RANDOM).

We will systematically review reports of RCTs published within two years in 25 general medical journals with the highest impact factor according to the Journal Citation Report (JCR)-2010 (Figure 1). Eligible trials are designed DUR trials without a limitation on the exact ratio. This study satisfied the ethical requirements of the Hamilton Health Sciences/Faculty of Health Sciences Research Ethics Board.

### Definitions

We define a DUR RCT to have pre-planned uneven numbers of participants in the experimental and control groups, including studies with more than two arms. We will contact the study authors to definitively establish if the study was *pre-planned* to have an uneven number of participants in experimental and control groups if published material, such as protocols or reports of the results, do not provide sufficient information.

If trial authors do not respond to our queries, two investigators will make independent judgments and reach consensus, if necessary with help from a third investigator. The criteria for the judgment will include: (1) for studies with total sample sizes of less than 1,000, if the randomization ratio was equal to or larger than 1.25; or (2) for studies with total sample size equal to or greater than 1,000, if the randomization ratio was equal to or larger than 1.05, we will consider them as DUR. Small changes in group size in small sample trials may have a higher impact on the ratio than in larger sample size trials. Thus, we decided to use different thresholds according to different sample sizes. We selected the 1.25 and 1.05 cut-offs in order to achieve a highly sensitive identification of DUR trials. The rationale, based on a preliminary literature review, was as follows. First, 7:6(1.17) [26] was the smallest ratio mentioned by authors of DUR trials, and 1.05 was a relatively low threshold. We assume that any trial with a ratio equal or below 1.05 has a less than 5% possibility of being a DUR trial. In our scoping exercise, we have not detected any trial with a ratio lower than 1.05 as a DUR trial or larger than 1.25 as a non-DUR trial. To calculate the randomization ratio, we used the larger group divided by the smaller one. For instance, if the sample size of the experimental group is 550, and the control group is 520, then 550/520 = 1.06; or if the experimental group’s sample size is 520, and the control group is 550, the ratio should still be 550/520 = 1.06.

We defined participation rate as the proportion of eligible individuals who participated; that is, number of eligible individuals (NEI) minus number of individuals declining to participate (NIDP) divided by number of eligible individuals (NEI):

Participation rate = (NEI-NIDP)/NEI

We will also calculate a recruitment rate as the proportion of participating individuals out of those actually approached in the trials as follows:

Recruitment rate = Participated individuals/Approached individuals.

### Eligibility criteria

1. RCT designed with an uneven randomization;

2. RCT assessing at least one patient important outcome, defined as one for which one would answer the following question with ‘yes’: ‘If the patient knew that this outcome was the only thing to change with treatment, would the patient consider receiving this treatment if it was associated with adverse effects, inconvenience, or cost?’ Such outcomes include mortality, morbidity, and outcomes reported by patients. We will consider surrogate outcomes (such as changes in blood pressure, HbA1c) as not important to patients [27];

3. Superiority trials (that is trials that are designed to demonstrate that one treatment is more effective than another).

We will exclude trails if they are (1) an N-of-1 RCT; (2) a RCT reported in a research letter; (3) reports of secondary analysis of previously published RCTs; (4) reports of subgroup analysis of previously published RCTs; (5) previously reported RCTs (for example, longer follow-up or a second trial included approximately the same amount of participants as the first one); (6) cross-over RCT; (7) factorial design; (8) cluster RCT.

When we are analyzing the impact of DURs on particular factor, such as participation rate, we will exclude (1) trials with more participants in the control group than in the experimental group, which is a ratio of participants in control versus experimental group larger than 1.25; or (2) trials omitting to describe obtaining informed consent from participants.

### Literature search

Eligible studies will be searched among the highest ranked 25 general medical journals according to the number of citations reported in the JCR by Thomson ISI (Institute for Scientific Information) in 2010: *New England Journal of Medicine (NEJM), Lancet, Journal of the American Medical Association (JAMA), Annals of Internal Medicine (AIM), PLOS Medicine, British Medical Journal (BMJ), Archives of Internal Medicine, Canadian Medical Association Journal (CMAJ), Journal of Internal Medicine, BMC Medicine, MAYO Clinic Proceedings (MCP), American Journal of Medicine (AJM), Annals of Family Medicine, Annals of Medicine, Medicine, American Journal of Preventive Medicine (AJPM), Cleveland Clinic Journal of Medicine (CCJM), Preventive Medicine (PM), British Medical Bulletin (BMB), American Journal of Managed Care (AJMC), Translational Research, Medical Clinics of North America (MCNA), Journal of General Internal Medicine (JGIM), European Journal of Clinical Investigation (EJCI), Medical Journal of Australia (MJA)*. Two journals, *Annual Review of Medicine* and *Cochrane Database Systematic Review*, which focus on review articles, were not included. We will search Medline (OVID interface) using the Cochrane Collaboration’s ‘highly sensitive’ search strategy to identify RCTs (Additional file 1).

### Review process

Working in pairs, reviewers will select studies (title/abstract screening and full-text screening) and abstract data, in duplicate and independently. Disagreements will be discussed and resolved between each pair of reviewers, if necessary with help of an arbitrator. Two investigators (DW, HJS) will serve as the arbitrators for all studies. We will use electronic forms for title and abstract screening, for full-text screening, and for data abstraction.

### Selection of studies

Reviewers will first screen the titles and abstracts of the identified citations for potentially eligible RCT reports. Then, they will screen full-texts of potentially eligible RCT reports using an electronic, standardized spreadsheet with corresponding written instructions. We will prepare detailed written instructions. Before extracting data from DUR trials, we will contact the study authors to clarify, if it was a DUR trial or if the unbalanced allocation was a product of chance.

### Sample size

Applying our search strategy to the years 2010 to 2011, we estimate that we will identify approximately 150 eligible DUR papers. For three plausible levels of participation rate, 25%, 50% and 75%, in non-DUR trials, with three respective minimal important increases of 5.3%, 10%, and 11.7% in DUR trials, a sample size of 150 would result in 95% confidence intervals (95% CIs) for those increases of (23% to 38%); (52% to 68%) and (81% to 92%), respectively. We determined the minimal important increase for three different levels of participation rate based on the responses of a brief survey amongst colleagues (Additional file 2).

In the regression analysis, the dependent variable will be the participation rate, and we estimate that ten independent variables, including the randomization ratio, will be included in the final model. It is widely accepted that one can use one independent variable per ten occurrences of the dependent variables in a linear regression analysis, which means that 150 eligible DUR trials will be sufficient for the regression analysis.

### Data abstraction

We will conduct a comprehensive search for all published material of each included DUR trial in order to gather all the available information about the participation rate. This material includes, for example, protocols, appendices, supplementary documents or other relevant published articles. However, for some of the RCTs we may not be able to gather sufficient information.

For all the reports of eligible DUR trials, we will extract information about the study design, methodological quality, and DUR-related data using a pilot-tested standardized data abstraction form (Additional file 3):

1. Design information

We extracted data on: number of study centers, ratio of randomization, clinical area, type of intervention, type of control, number of arms, total number of randomized participants, planned sample size and primary and secondary outcomes. We will also record measures to encourage recruitment (for example, telephone reminder).

2. Methodological quality

We will determine the methodological quality by abstracting the following factors: blinding, allocation concealment, premature discontinuation and reason for stopping. We will use ‘definitely yes/probably yes/probably not/definitely not’ as judgment options in the data abstracting of blinding [28], and will determine the category of concealment of allocation (see Additional file 3 for more details).

3. DUR data

We will collect reasons for DUR, reported baseline characteristics of those non-participants (yes/no), as well as number of non-participants due to different reasons when reported by the trial. We divided the reasons for those choosing to *Not Participate* into three types, that is, (1) *Subjective reason*: *not to participate* was due to subjective reasons, such as not interested; (2) *Unclear reason*: reasons that were reported as ‘unclear’ or not reported; and (3) *Other reason*: all the reasons in between, we consider as *Other reasons*. Possible descriptions of the reasons and the corresponding categories are listed in Additional file 4.

Information as number of authors contacted, type of consent (opt-out or opt-in), duration of recruitment, type of randomization ratio, report of CONSORT flow diagram and financial incentives for participants will be abstracted.

4. Others

We will identify data that is inconsistent in the report (for example, differences between the results section and tables), as well as factors reported by the authors that might impact on the participation rate.

If the reasons for persons refusing to participate or if the number of eligible persons were not clearly or completely reported, we will provide study authors with the opportunity to confirm or refute the abstracted data by contacting and sending them the key results of our data abstraction, such as number of persons refusing to participate due to different reasons.

### Data analysis

#### Descriptive of the data

We will report the design information of DURs, number of study centers, clinical areas that were covered, type of intervention, type of control, number of arms, planned sample size, the outcomes and measures to encourage recruitment.

Methodological quality of DURs will be reported for several factors: type of blinding, allocation concealment, trials stopped early and the reasons for stopping.

#### Reporting of DUR information

We will calculate the proportion of RCTs that are DUR trials using the total number of RCTs as the denominator for each publication year (2010 and 2011). We will also calculate the proportions of DUR trials (1) reporting a CONSORT flow diagram; (2) reporting details about the informed consent procedure; (3) reporting UNCLEAR as reason of non-participation; (4) reporting reasons for using DUR.

For the proportion of studies where there is residual uncertainty about whether or not they were DUR trials, that is, we cannot obtain any information from the published materials or any response from the authors and the randomization ratio is between 1.05 and 1.25, we are planning a sensitivity analysis of this group of trials despite their sample sizes. All the above calculations of DUR trails will also be reported based on this sensitivity analysis.

#### Reporting of participation rate

We will report the participation rate for DUR trials. If the number of eligible individuals was not reported, and no further information could be obtained from the authors, meaning that we will be unable to determine the participation rate, we will use a median participation rate of the other studies in the same clinical area and perform a sensitivity analysis.

#### Methods used for analyzing reported participation rate

The unit of analysis will be the individual trial. We will conduct a linear regression analysis to estimate the association between the randomization ratio and the percentage of non-participation due to subjective reasons. The dependent variable in this analysis will be the participation rate. Our main predictor of interest is the randomization rate. We will also include type of informed consent, clinical area, type of design, number of arms, measures to encourage recruitment, duration of recruitment, and financial incentives for participants as independent variables.

In addition, we will perform an analysis considering the influence of the randomization ratio on the overall non-participation rate.

#### Subgroup analysis

We will perform subgroup analysis on (1) patient-reported or no patient-reported outcomes; (2) single or multiple experimental arms, to determine which factors were more likely to impact the participation rate.

## Discussion

This protocol describes a methodological study aiming to evaluate the epidemiology and to explore factors associated with DUR. Strengths of our study protocol include a systematic literature search with the goal of compiling a comprehensive sample of DUR trials, and the transparent and systematic methods used with respect to selecting eligible studies and abstracting data.

We will focus on reports of trials published in the top 25 general medical journals that are more likely to influence clinical practice than those published in lower profile journals. Moreover, RCTs published in these major medical journals are typically of higher methodological quality [29], and tend to report more detailed information on reasons for non-participation than studies with lower impact profile. However, the representativeness of our study may be limited because of the decision to focus on high impact journals.

There are several potential reasons for using DUR. In RCTs with a fixed budget, DURs may increase the power of the trial by allowing more participants to be recruited in the least expensive trial arm [30]. Also, DUR may have ethical advantages by reducing the number of subjects exposed to a potentially dangerous treatment [16], as well as by increasing the number of subjects who would be randomized to what was thought to be the superior therapy [15]. Encouraging patient enrollment and increasing compliance in a placebo-controlled trial are other reasons for DUR [31]. Moreover, unequal allocation is commonly used to increase the probability of success in multi-stage adaptive design studies [32].

Although the power of the *t*-test for the difference in means of two groups is maximized with equal sample sizes [33], Lachin [34] pointed out that the gain in power is trivial. For large trials (total n > 200), the susceptibility of a randomization procedure to such imbalances appears not be of statistical concern. Additionally, McEntegart and Dawson [35] commented that maximizing power for pairwise comparisons is a reason for unequal allocation in dose-response studies.

The accuracy of our results will depend on the number of DUR trials we will identify and the number of other factors we will abstract; for example, individuals who decline to participate even when they are eligible. Though the CONSORT statement [36] recommends that authors give detailed information about those who were excluded due to a variety of reasons, the available information indicated that inadequate reporting of non-participation is a greater problem than anticipated [37]. In this study, without a concurrent control group of pre-planned even randomization ratio (1:1) trials, we cannot quantitatively estimate the effect of DUR on a specific factor. On the basis of the result of our study, we may be able to do further research to compare the relative risk (RR) of a specific factor which is closely associated with DRU with the RR from matching non-DUR trials.

It is very likely that investigators, journal editors, and clinical experts remain unaware of the problems associated with inadequate participant recruitment, ranging from inconclusive numbers to possible alteration of the study results [37]. Hunninghake and his colleagues [37] noted that accrual rates are often not clearly reported. Also, they were unable to estimate how many studies were terminated or yielded inconclusive results because of inadequate participant recruitment.

Findings of our study may influence recommendations on how detailed information should be included when the number of participants is reported. Through rigorous search strategies (with the goal of compiling a comprehensive sample of RCTs with uneven randomization, and independent evaluation of all the eligible RCTs) and statistical analysis with sufficient power, we could provide useful information of the epidemiology and factors, for example, participation rate, associated with DUR. Evidence of vulnerability of participation recruitment will call for improving study design and trial implementation to increase participation rate. Our study should make a significant contribution to the methodology of RCTs and provide guidance for trialists and those interpreting RCTs.

## Abbreviations

DUR: Designed uneven randomization; NEI: Number of eligible individuals; NIDP: Number of individuals declining to participate; RCTs: Randomized controlled trials.

## Competing interests

The authors declare that they have no competing interests.

## Authors’ contributions

HJS, DW, EAA, GHG, PJD and MB contributed substantially to the design of this study. NS, DW, HJS and RAM established the literature search strategy. DW, RBP, NS, RAM, BP, KP, NP, TL, YZ and MF contributed to the study design and will review articles and collect data during the study. QZ and DW conducted the survey of sample size calculation and done the calculation. DW and HJS will supervise the process of study. DW and HJS drafted the manuscript in close collaboration with the other authors. All authors read and approved the final manuscript.

## Supplementary Material

Additional file 1 Search strategy for Medline using OVID interface. Click here for file

Additional file 2 Disclosure form of the survey on Minimal Important Difference. Click here for file

Additional file 3 Data Abstraction Form. Click here for file

Additional file 4 Examples of each type of non-participation reasons. Click here for file
