# Supplementary material for: Methodological survey of designed uneven randomization trials (DU-RANDOM): a protocol
Source: Trials. 2014 Jan 23;15:33. doi: 10.1186/1745-6215-15-33 (PMC3902027; doi:10.1186/1745-6215-15-33)
Supplement: Additional file 1 — Search strategy for Medline using OVID interface. [file 1745-6215-15-33-S1.doc]

**Additional file 1:** Search strategy for Medline using OVID interface

1     "new england journal of medicine".jn. (22351)
2     lancet.jn.(33572)
3     jama.jn. (18994)
4     "annals of internal medicine".jn. (8598)
5     "plos medicine public library of science".jn. (1902)
6     bmj.jn. (35728)
7     "archives of internal medicine".jn. (6892)
8     cmajcanadian medical association journal.jn. (9346)
9     "journal of internal medicine".jn. (2363)
10    bmcmedicine.jn. (466)
11   mayo clinic proceedings.jn. (3602)
12     "american journal of medicine".jn. (6149)
13     "annals of family medicine".jn. (924)
14     "annals of medicine".jn. (1169)
15     medicine.jn. (590)
16     or/1-15 (152646)
17     "american journal of preventive medicine".jn. (3199)
18     "cleveland clinic journal of medicine".jn. (2053)
19     preventive medicine.jn. (2972)
20     british medical bulletin.jn. (831)
21     "american journal of managed care".jn. (2198)
22     "translational research the journal of laboratory & clinical medicine".jn. (492)
23     "medical clinics of north america".jn. (1169)
24     "journal of general internal medicine".jn. (3602)
25     "european journal of clinical investigation".jn. (2541)
26     "medical journal of australia".jn. (8935)
27     randomized controlled [trial.pt](http://trial.pt/). (227274)
28     controlled clinical [trial.pt](http://trial.pt/). (38385)
29     randomized.ab.(183810)
30     placebo.ab.(86995)
31     randomly.ab.(126765)
32     trial.ab.(178261)
33     clinical trials as topic.sh.(76444)
34     or/27-33 (568309)
35     exp animals/ not humans.sh. (1541157)
36     34 not 35 (515015)
37     or/17-26 (27992)
38     16 or 37 (180638)
39     38 and 36 (16205)
40     limit 39 to yr="2010 - 2011" (1953)
