# Supplementary material for: Methodological survey of designed uneven randomization trials (DU-RANDOM): a protocol
Source: Trials. 2014 Jan 23;15:33. doi: 10.1186/1745-6215-15-33 (PMC3902027; doi:10.1186/1745-6215-15-33)
Supplement: Additional file 2 — Disclosure form of the survey on Minimal Important Difference. [file 1745-6215-15-33-S2.doc]

**Additional file 2:** Disclosure form of the survey on Minimal Important Difference

**Sample size calculation for the DU-RANDOM (Designed Uneven Randomization) project**

What’s already known on the topic?

1. Sample size in randomized trials is increased in designed uneven randomized (DUR) trials compared with non-DUR trials

The smaller the control event rate or relative risk reduction (RRR), the larger is the difference in sample size between DUR and non-DUR trials.

Attached are figures of total sample size under different control rate and different RRR (5%, 20%, 40%, 50% and 60% respectively) with 1:1, 2:1, 3:1 and 4:1 ratios of randomization.

1. Cost is reduced when using DUR in clinical trials if the experimental treatment is cheaper (an example from reference 1)[[1]](#endnote-2)[[2]](#endnote-3)[[3]](#endnote-4)

For instanceError: Reference source not found, T_1_, a new treatment for hypertension, costs $16 per therapeutic course while conventional therapy (T_2_) costs $400.

If the standard deviation of each measured decrease in diastolic blood pressure is σ_1_=σ_2_=5mmHg in each group, and if α=0.05, β=0.01, then the non-DUR trial would require a sample size of 22 subjects in each group, that is n_1_=n_2_=22; the total cost for the non-DUR trial would be 22🞨16+22🞨400=$9152.

For a DUR trial, the total sample size would be 79 subjectsError: Reference source not found, if the randomization ratio for T_1_ versus T2 = 5:1. The total cost of DUR trial=65🞨16+13🞨400=$6240.

1. Besides saving cost, other reasons for using DUR in clinical trials include avoiding risk (e.g. toxicity)[[4]](#endnote-5) but primarily to **encourage patient enrolment2**[[5]](#endnote-6)

. ……………………………………………………………………………………………

Based on these examples, background and your own experience, what is your opinion about how different the participation rate in DUR trials should be compared to non-DUR trials (say for a 2:1 compared with a 1:1 ratio) in terms of relative and absolute increase?

*For each of the participation rate, please provide the absolute and/or relative difference that you find is noteworthy (in percentage).*

| **The baseline participation in a 1:1 trial would be** | **What is the minimal increase in participation in a 2:1 randomized trial that you find to represent a noteworthy difference (please give percentages)** | | **Comments?** |
| --- | --- | --- | --- |
|  | **Absolute *difference*** | **Relative *difference*** |  |
| **25%** |  |  |  |
| **50%** |  |  |  |
| **75%** |  |  |  |

1. Gail M, Williams R, Byar DP, Brown C. How many controls? J Chronic Dis. 1976;29(11):723-31 [↑](#endnote-ref-2)
2. Singer J. Estimating sample size for continuous outcomes, comparing more than two parallel groups with unequal sizes. Stat Med. 1997;16(24):2805-11. [↑](#endnote-ref-3)
3. A L Avins. Can unequal be more fair? Ethics, subject allocation, and randomised clinical trials. J Med Ethics. 1998; 24(6): 401–408. [↑](#endnote-ref-4)
4. Sahai H, Khurshid A. Formulae and tables for the determination of sample sizes and power in clinical trials for testing differences in proportions for the two-sample design: a review. Stat Med. 1996;15(1):1-21. [↑](#endnote-ref-5)
5. Schouten HJ. Sample size formula with a continuous outcome for unequal group sizes and unequal variances. Stat Med. 1999;18(1):87-91. [↑](#endnote-ref-6)
