# Supplementary material for: Methodological survey of designed uneven randomization trials (DU-RANDOM): a protocol
Source: Trials. 2014 Jan 23;15:33. doi: 10.1186/1745-6215-15-33 (PMC3902027; doi:10.1186/1745-6215-15-33)
Supplement: Additional file 3 — Data Abstraction Form. [file 1745-6215-15-33-S3.doc]

**Additional file 3:** Data Abstraction Form

**D**esigned **U**neven **Random**ization **(DU-RANDOM)** project

***Data abstraction Form***

**Screener initials: Study ID: Author, year: ___________________, _______**

**Journal:**

⁪01=AJMC ⁪02=AJM ⁪03=AJPM ⁪04=AFM ⁪05=AIM

⁪06=AM ⁪07=AI ⁪08=BMC MED ⁪09=BMB ⁪10=BMJ

⁪11=CMAJ ⁪12=CCJM ⁪13=EJCI ⁪14=JGIM ⁪15=JIM

⁪16=JAMA ⁪17=LANCET ⁪18=MCP ⁪19=MCNA ⁪20=MJA

⁪21=MEDICINE ⁪22=NEJM ⁪23=PLOS M ⁪24=PREV M ⁪25=TR

1. **Eligible RCT? ⁪**⁪ No, *type of RCT:* ⁪**Exclude***, stop here*

⁪ ⑴N-of-1 RCT ⁪ ⑶Cluster trial

⁪ ⑵Factorial design ⁪ ⑷Cross over study

⑸Reports of secondary or subgroup analysis/reported in a letter

⁪ Yes, *type of RCT:*

⁪ ⑴Two arms-parallel design ⁪ ⑵Multiple Arms-parallel design

1. **Eligible uneven RCT?** ⁪ No ⁪**Exclude***,* *stop here*

⁪ Yes

⁪ None *specified*

Response from the author

⁪ No ⁪ **Exclude**, stop here

⁪ Yes, but not pre-planned ⁪ **Exclude**, stop here

⁪ Yes, pre-planned

No response

1. **Trial described as:** ⁪ Non-inferiority ⁪**Exclude**

Equivalence ⁪**Exclude**

⁪ Superiority

⁪ Neither*, list details:* ___________________________

1. **Is the primary outcome a patient important one?**

⁪ No*, list details*: ___________________________ ⁪ **Exclude**

⁪ Yes, *list details*: ___________________________

**If exclude, reason for exclusion:**

⁪ ⑴Not eligible RCT

⁪ ⑵Not eligible uneven RCT

⁪ ⑶Non-inferiority or Equivalence

⁪ ⑷No participant’s informed consent

⁪ ⑸Outcome not patient important

⁪ ⑹Other:

*Please fill out this box for each study*

**⁪ Include in DU-RANDOM**

**⁪ Exclude from DU-RANDOM**

**⁪ 3^rd^ reviewer needed** *(no consensus between 2 reviewers)*

| **Background information** | | | |
| --- | --- | --- | --- |
|  | **Number of study centers** | n = | |
|  | **Time period of recruitment** | - Month: ________________________________ - Not reported | |
|  | **Preplanned randomization ratio**  *Check all that Apply* | - ⑴Reported, list details: ________________________________ - ⑵Response from the author, list details: ___________________ - ⑶Not reported - ⑷no response from the author | |
|  | **Design type of RCT** | - Open - Blinded - Other, list details: ___________________________ | |
|  | **Funding**  *Check all that Apply* | - ⑴Private for profit, industry only supplying medication - ⑵Private for profit, other - ⑶Private not for profit - ⑷Governmental - ⑸Not funded - ⑹Not reported | |
|  | **Reason for DU-RANDOM**  *Check all that Apply* | - ⑴Cost - ⑵Expected drop-outs - ⑶Patient acceptability - ⑷Ethics - ⑸Gaining experience of treatment - ⑹Other, list details: _______________________________________ - ⑺Not stated | |
|  | **Patients received compensation**  *Check only one* | - Not reported - None - Yes, list details:________________________________________ | |
|  | **Informed consent obtained?** | - Not reported - No - Yes————**┑**   Procedure of informed consent::   - Not reported - Opt-out procedure - Opt-in procedure - Other, list details: _______________ | |
|  | **Preplanned sample size calculation**  *Check only one* | - Yes - No - Not reported | |
|  | **Measures to encourage recruitment** | - None - Yes, telephone reminder - Yes, others, list details: _______________________________ | |
|  | **Clinical area**  *Check only one* | **Medical**   - ⑴Dermatology - ⑵Cardiology - ⑶Endocrinology - ⑷Gastro Intestinal - ⑸Hematology - ⑹Intensive Care - ⑺Infectious Diseases - ⑻Neurology - ⑼Oncology - ⑽Psychiatric - ⑾Renal - ⑿Respiratory - ⒀Rheumatology - ⒁Other *(specify)*: | **Surgical**   - ⑴Cardiac surgery - ⑵General surgery - ⑶Obstetrics/ Gynecology - ⑷Ophthalmology - ⑸Orthopedic surgery - ⑹Otorhinolaryngology (ENT: Ear Nose Throat) - ⑺Neurosurgery - ⑻Plastic surgery - ⑼Thoracic surgery - ⑽Urologic surgery - ⑾Vascular surgery - ⑿Other *(specify)*: |
|  | **Intervention**  *Check only one* | - ⑴Pharmacological - ⑵Surgery/ Invasive procedure - ⑶Rehabilitation - ⑷Behavioral intervention | - ⑸**Complementary and alternative medicine** *(specify)*: - ⑹Lifestyle modification - ⑺Other *(specify)*: |
|  | **Control**  *Check only one* | - ⑴Standard care - ⑵Placebo - ⑶Pharmacological - ⑷Surgery/ Invasive procedure - ⑸Rehabilitation - ⑹Behavioral intervention | - ⑺**Complementary and alternative medicine** *(specify)*: - ⑻Lifestyle modification - ⑼Waiting list - ⑽Other *(specify)*: |

| **Methodological quality** | | | | | | | |
| --- | --- | --- | --- | --- | --- | --- | --- |
|  | **Concealment of Allocation**  *Check only one* | - ⑴Sequentially numbered, opaque, sealed envelope | | - ⑵Coded medication containers | | - ⑶Central randomization (e.g. by telephone) | |
|  |  | - ⑷Envelopes, other | | - ⑸Open random allocation schedule | | - ⑹Quasi-randomized | |
|  |  | - ⑺ “Concealed”, no method described | | - ⑻“Not concealed” | | - ⑼Not reported | |
|  | **Blinding of patients** | - Definitely yes | - Probably yes | | - Probably not | | - Definitely not |
|  | **Blinding of health Care providers** | - Definitely yes | - Probably yes | | - Probably not | | - Definitely not |
|  | **Blinding of data collectors** | - Definitely yes | - Probably yes | | - Probably not | | - Definitely not |
|  | **Blinding of outcome adjudicators** | - Definitely yes | - Probably yes | | - Probably not | | - Definitely not |
|  | **Blinding of data analysts** | - Definitely yes | - Probably yes | | - Probably not | | - Definitely not |
|  | **Study stopped early for benefit** | - Yes **──┑**   ⑴A planned intermediate analysis?   - Yes - No   ⑵A stopping rule predetermined?   - Yes - No | | | - No | |  |

| **du-random data** | | | |
| --- | --- | --- | --- |
|  | **Number of** persons **contacted** | - Reported, **(skip to 27)**   List the number **____________** | - Not reported |
|  | **Need to ask authors for numbers screened** | - Yes | - No, list details: ________ |
|  | **Number of eligible participations** | - Reported,   List the number **____________** | - Not reported |
|  | **Need to ask authors for numbers of eligible participations** | - Yes | - No, list details: ________ |
|  | **Number of persons in experimental group** | - List the number ____________ |  |
|  | **Number of persons in control group** | - List the number ____________ |  |
|  | **Number of Intervention group**  *Circle one choice* | 1 2 3 4 5 6 7 8 9 10 | |
|  | **Number of control group**  *Circle one choice* | 1 2 3 4 5 6 7 8 9 10 | |
|  | **Baseline characteristics of non-participation** | - Reported | - Not reported |
|  | Number of persons refused to participate due to SUBJECTIVE reasons: | - Reported,   List the number **____________** | - Not reported **(skip to q36)** |
|  | Reported Terms of SUBJECTIVE reasons:  *Check all that apply* | - ⑴Withdrew consent/consent withdrawn - ⑵Decline consent/consent declined - ⑶Decline to participate/declined invitation - ⑷Declined - ⑸Refused/Refused to participate/didn’t want to participate - ⑹Declined randomization - ⑺Abandoned/changed mind/not interested - ⑻Other (specify): ___________________________ | |
|  | **Need to ask authors for the number of persons refused to participate** | - Yes | - No, list details: ________ |
|  | **Number of persons refused to participate due to OTHER reasons:** | - Reported,   List the number **________** | - Not reported **(skip to q39)** |
|  | **Reported Terms of OTHER reasons:**  *Check all that apply* | - ⑴Not meet eligibility (inclusion/exclusion) criteria - ⑵No physician approval - ⑶Medical reasons - ⑷Incomplete screening packages - ⑸Participated in another or previous trial - ⑹Adverse event - ⑺Distance/Travel - ⑻Family issues/Busy/ Schedule conflict - ⑼Non compliance - ⑽Unable to give consent - ⑾Others (specify): ___________________________ | |
|  | **Number of persons refused to participate due to UNCLEAR reasons:** | - Reported,   List the number **________** | - Not reported **(skip to q41)** |
|  | **Reported Terms of UNCLEAR reasons:**  *Check all that apply* | - ⑴“Non-medical reasons” - ⑵“Unclear” - ⑶“Other” (as a term) - ⑷“Lost to follow-up” - ⑸No exact term could be found - ⑹Other (specify): ____________________________ | |

|  | **Inconsistency in Participation Rate (PR) data** *(explain below)*  **□No inconsistency data □Yes, list details:___________________** |
| --- | --- |
|  | **Misunderstanding of PR or questionable inferences (***explain below)*  **□No question □Yes, list details:___________________** |
|  | **Time required to complete this form: ______ min** |
