# Supplementary material for: Methodological survey of designed uneven randomization trials (DU-RANDOM): a protocol
Source: Trials. 2014 Jan 23;15:33. doi: 10.1186/1745-6215-15-33 (PMC3902027; doi:10.1186/1745-6215-15-33)
Supplement: Additional file 4 — Examples of each type of non-participation reasons. [file 1745-6215-15-33-S4.doc]

**Additional file 4:** Examples of each type of non-participation reasons

**SUBJECTIVE reasons:**

Withdrew consent/consent withdrawn;

Decline consent/consent declined;

Decline to participate/declined invitation;

Withdrew;

Declined;

Refused;

Refused to participate/didn’t want to participate;

Declined randomization/didn’t want to be randomized;

Abandoned/changed mind;

Not interested.

OTHER reasons:

Did not meet eligibility (inclusion/exclusion) criteria;

No physician approval;

Medical reasons;

Incomplete screening packages;

Participated in another or previous trial;

Adverse event;

Distance;

Travel;

Family issues;

Busy;

Schedule conflict;

Non compliance;

Could not meet study demands;

Were too active;

Unable to give consent.

UNCLEAR reasons:

Non-medical reasons;

Could not be contacted;

Other.
